# Correction: The selective hydrosilylation of norbornadiene-2,5 by monohydrosiloxanes

**DOI:** 10.1039/c9ra90079a

**Published:** 2019-10-30

**Authors:** Marina A. Guseva, Dmitry A. Alentiev, Evgeniya V. Bermesheva, Ilya A. Zamilatskov, Maxim V. Bermeshev

**Affiliations:** A. V. Topchiev Institute of Petrochemical Synthesis of Russian Academy of Sciences 29 Leninsky Prospekt 119991 Moscow Russia d.alentiev@ips.ac.ru; I. M. Sechenov First Moscow State Medical University 8 Bld. 2 Trubetskaya Str. 119991 Moscow Russia; A. N. Frumkin Institute of Physical Chemistry and Electrochemistry of Russian Academy of Sciences 31 Bld. 4 Leninsky Prospekt 119071 Moscow Russia; MIREA – Russian Technological University (M.V. Lomonosov Institute of Fine Chemical Technologies) 86 Vernadskij Prospect Moscow 119571 Russia

## Abstract

Correction for ‘The selective hydrosilylation of norbornadiene-2,5 by monohydrosiloxanes’ by Marina A. Guseva *et al.*, *RSC Adv.*, 2019, **9**, 33029–33037.

The authors regret that affiliation d was missing in the original manuscript. The correct affiliations are as presented here.

The Royal Society of Chemistry apologises for these errors and any consequent inconvenience to authors and readers.

## Supplementary Material

